# Role of Platelet-Rich Plasma Injection in Anterior Cruciate Ligament Reconstruction: A Meta-Analysis of Randomized Controlled Trials

**DOI:** 10.3390/bioengineering13040455

**Published:** 2026-04-13

**Authors:** Ahmed Abdirahman Ibrahim, Michael Opoku, Abakar Mahamat Abdramane, Mingqing Fang, Xu Liu, Abdulraheem Mustapha, Yusheng Li, Wenfeng Xiao, Kai Zhang, Shuguang Liu

**Affiliations:** 1 Department of Orthopaedics, Xiangya Hospital, Central South University, Changsha 410008, China; 248119013@csu.edu.cn (A.A.I.); michael_opuku@csu.edu.cn (M.O.); 248119012@csu.edu.cn (A.M.A.); 8303200916@csu.edu.cn (M.F.); 228112308@csu.edu.cn (X.L.); liyusheng@csu.edu.cn (Y.L.); xiaowenfeng@csu.edu.cn (W.X.); 2 Xiangya School of Medicine, Central South University, Changsha 410083, China; 3 Department of Cardiovascular Surgery, The Second Xiangya Hospital of Central South University, Changsha 410011, China; aamustapha21@csu.edu.cn; 4 National Clinical Research Center for Geriatric Disorders, Xiangya Hospital, Central South University, Changsha 410008, China; 5 Department of Orthopaedics, The First People’s Hospital of Changde City, Central South University, Changde 415000, China; 6 Department of Joint Surgery, Honghui Hospital, Xi’an Jiaotong University, Xi’an 710061, China

**Keywords:** platelet-rich plasma, anterior cruciate ligament reconstruction, clinical outcomes, image evaluation, meta-analysis

## Abstract

**Purpose:** To critically evaluate the role or effect of platelet-rich plasma (PRP) in anterior cruciate ligament (ACL) reconstruction in terms of clinical and radiological outcomes. **Method:** We conducted a systematic search of PubMed, Embase, the Cochrane Library, and Web of Science to identify relevant studies. Clinical outcomes included the Visual Analogue Scale (VAS), International Knee Documentation Committee (IKDC) subjective and objective evaluations, Lysholm score, Tegner score, anterior knee laxity, Knee Injury and Osteoarthritis Outcome Score (KOOS), Kujala score, Victorian Institute of Sport Assessment (VISA) scale, proprioception, isokinetic strength, and physical examination tests (anterior drawer, Lachman, and pivot-shift tests). Radiological outcomes encompassed measures obtained via magnetic resonance imaging (MRI), computed tomography (CT), X-ray, and ultrasound. Statistical significance was defined as a *p* value < 0.05, and all analyses were performed using R software (version 4.1.3). **Results:** A total of 23 studies, including 19 randomized controlled trials, met the inclusion criteria, encompassing 1072 patients overall. The meta-analysis showed significant differences between PRP group and non-PRP group with regard to VAS score at 6- and 12-month follow-up, Lysholm score at 6-month follow-up, and Tegner score at 6-month follow-up. Meta-regression showed that the two group differences in VAS score changed significantly with follow-up time (*p* < 0.01). In terms of radiological findings, about half of the assessments favored PRP to facilitate the graft maturation and integration at 6-month follow-up. **Conclusions:** PRP application in ACL reconstruction compared with non-PRP, may produce short-term but not long-term clinical outcomes such as VAS score, Lysholm score and Tegner score. While some short-term statistical differences exist, their magnitude and durability do not yet justify routine clinical adoption of PRP in ACL reconstruction. Larger samples and higher-quality studies are needed to support our results and further explore the advantages of PRP in other aspects. Level of evidence: Level II.

## 1. Introduction

Anterior cruciate ligament (ACL) rupture is among the most frequent knee ligament injuries, especially in young, physically active individuals participating in both competitive and recreational sports [1,2]. The ACL plays a critical role in maintaining knee stability by restraining excessive anterior tibial translation and rotational movements. In the United States alone, more than 250,000 ACL injuries are reported each year [3]. If untreated, ACL rupture may lead to recurrent instability, pain, functional impairment, reduced activity, and an increased risk of secondary meniscal or cartilage injury, potentially contributing to knee osteoarthritis [4,5,6,7]. Young, active patients often have high expectations for functional recovery and return to sport, making effective management essential [8].

Arthroscopic anterior cruciate ligament reconstruction (ACLR) is considered the gold-standard surgical intervention for restoring knee stability and enabling patients to return to their pre-injury level of activity [9,10,11,12]. The procedure involves replacing the torn ligament with a tissue graft, which serves as a biological scaffold to support ligament regeneration [13]. Despite advances in graft choice, fixation, and rehabilitation, some patients experience complications such as pain, delayed graft maturation, tunnel widening, or incomplete recovery [14], and only around 55% return to competitive sports [15]. Revision ACLR shows higher failure rates, three to four times that of primary reconstruction [16,17]. Consequently, enhancing biological healing through orthobiologic and other augmentation strategies has become a key focus of research [18,19].

Platelet-rich plasma (PRP) has emerged as a widely used therapeutic approach for various musculoskeletal conditions, including cartilage defects in knee osteoarthritis, tendinopathy such as lateral epicondylitis, and to enhance tendon-to-bone healing following arthroscopic rotator cuff repair [20,21,22]. PRP is derived from autologous blood and contains a concentrated mixture of growth factors and cytokines, including vascular endothelial growth factor (VEGF), insulin-like growth factor (IGF), fibroblast growth factor (FGF), platelet-derived growth factor (PDGF), transforming growth factor-β (TGF-β), epidermal growth factor (EGF), as well as PF4 and CD40L [23,24]. These biological mediators can promote angiogenesis, tissue remodeling or regeneration, and wound healing and function in regenerative medicine [24,25]. The use of PRP in ACL reconstruction has been controversial due to limited evidence on its efficacy, proper preparation method, injection doses and timing, and administration sites [26,27]. de Andrade A.L.L et al. [28] concluded in a systematic review and meta-analysis that PRP application in ACL reconstruction did not show any significant improvement in objective outcomes and less tunnel widening but rather showed little improvement in outcomes such as Lysholm score, Visual Analogue Scale (VAS), and knee laxity. Their findings did not favor PRP use in ACL reconstruction due to limited evidence. Davey, Martin S et al. [29] reported that PRP application in seven out of thirteen randomized controlled trials showed no clinical improvement in functional and patient-reported outcomes following ACL reconstruction. They found two studies evaluating PRP for autologous hamstring showing significant improvement in MRI findings.

In light of this, we conducted a meta-analysis incorporating a more rigorous selection of randomized controlled trials to provide a more precise evaluation of the role of platelet-rich plasma (PRP) in anterior cruciate ligament (ACL) reconstruction. This approach aimed to generate more robust and reliable conclusions. Accordingly, the objective of this study was to critically assess the effects of PRP application in ACL reconstruction with respect to both clinical and radiological outcomes.

## 2. Methods

Our meta-analysis was reported following the PRISMA (Preferred Reporting Items for Systematic reviews and Meta-Analyses) guidelines and is presented in Supplementary Table S1 [30]. The protocol and considerations of this systematic review and meta-analysis were registered in the International Prospective Register of Systematic Reviews (PROSPERO) on 14 November 2024, ID: CRD42024608614, with all registered information and primary outcomes adhering to in the original manuscript.

### 2.1. Literature Search

Two authors independently conducted systematic searches of the following electronic databases: PubMed, Cochrane Library, Embase, and Web of Science. They identified articles published from database inception through 28 October 2024. To ensure comprehensive coverage and minimize the risk of omitting newly published studies, an additional updated search was subsequently performed on 23 December 2025. The search strategies employed a combination of entry words and Medical Subject Headings (Mesh) terms, focusing on key terms or phrase: (“anterior cruciate ligament” OR “anterior cruciate ligament reconstruction” OR “anterior cruciate ligament injuries”) AND (“platelet-rich plasma” OR “platelet-rich fibrin” OR “plasma rich in growth factors” OR “platelet-derived growth factors” OR “platelet gel” OR “platelet concentrate” OR “platelet lysate” OR “platelet supernatant”), with limitation to English articles. To augment the comprehensiveness of our search, supplementary searches were undertaken by examining articles included in systematic reviews as well as reference lists in other relevant articles, to identify studies not initially retrieved from the databases.

### 2.2. Study Eligibility

The obtained literature was first de-duplicated. The titles and abstracts of the remaining papers were then separately screened by two authors while closely following predetermined inclusion and exclusion criteria. In order to find pertinent studies that met the predetermined criteria, a manual screening procedure was carried out on the references obtained from systematic reviews concurrently. Each qualified work of literature was then obtained in its entirety to determine whether or not it should be included. In order to reach a consensus, the two authors deliberated over differences in the literature they chose. A third author made the final choice if debates continued.

### 2.3. Inclusion Criteria

The inclusion criteria of this meta-analysis were as follows:

(1) Full-text, peer-reviewed articles written in English; (2) randomized controlled trials (RCTs); (3) studies examining primary ACL reconstruction with PRP versus a control group in which patients who had ACL reconstruction alone; (3) at least 1 of the selected outcomes was reported: clinical outcomes (VAS score, knee anterior laxity, patient-reported outcomes, physical examination for anterior cruciate ligament injuries, muscle strength, or knee proprioception) or radiographic outcomes (graft maturation and integration, tunnel widening, or harvest site healing). VAS score, IKDC, Lysholm, and Tegner scores were predefined as the primary clinical outcomes, with the 12-month follow-up designated as the primary end point, as this interval was the most consistently reported across the included studies and reflects a clinically meaningful window for functional recovery after ACL reconstruction. Outcomes assessed at earlier (3 months) or later follow-ups (12–24 months), as well as other clinical and radiological endpoints, including KOOS, Kujala score, isokinetic muscle strength, proprioception, physical examination measures, anterior knee laxity, and graft maturation, were considered secondary or exploratory outcomes.

The decision to conduct meta-analyses was based on predefined criteria, including the availability of comparable outcome measures reported in at least two studies with sufficient statistical data. This approach was implemented to ensure methodological transparency and to avoid any impression that outcome selection was performed post hoc.

### 2.4. Exclusion Criteria

The exclusion criteria of this meta-analysis were as follows: (1) non-clinical studies such as in vitro or animal studies; (2) studies where the full text of the literature was not available; (3) reviews, letters, and conference abstracts; and (4) studies including patients with revision surgery or previous knee injuries.

### 2.5. Data Extraction

Using a structured literature information table, two authors independently extracted data from the finalized included literature. When disagreements arose, the first attempt was to reach a compromise through negotiation; if this failed, the decision was forwarded to a third author. The following elements were included in the extracted data: (1) fundamental features of the literature, such as the first author and the year of publication; (2) experimental information, such as study design, degree of evidence, and surgical techniques (such as graft type, source, fixation technique, and rehabilitation protocol); (3) patient information: number of patients in each group, number of missing follow-up, gender ratio, age, and duration of follow-up; (4) preparation and application of platelet-rich plasma: processing machine, whole blood volume, anticoagulant, spin speed and time, activation agent, platelet concentration, PRP form, application location, volume, and time points; (5) outcomes as previously mentioned.

### 2.6. Quality Assessment

To evaluate the risk of bias in RCTs, the authors used the Cochrane Collaboration’s risk of bias tool [31]. In the beginning, disagreements were supposed to be settled by negotiation between the two writers (MO AND MQF); if that did not work out, a third author (AAI) was consulted. The Seven items are included in the Cochrane scale: (1) random sequence generation; (2) allocation concealment; (3) blinding of participants and personnel; (4) blinding of outcome assessment; (5) Incomplete outcome data; (6) selective reporting; (7) other bias. Each item was categorized as low risk, high risk, or unclear.

### 2.7. Statistical Analysis

All data analyses were conducted using the metan package in R (version 4.1.3). For clinical outcomes, such as VAS score, IKDC score, Lysholm score, and Tegner score, data were synthesized using means and standard deviations, or medians and quartiles. Medians and quartiles were converted to means and standard deviations using the Hozo’s method and Bland’s method, respectively. The inverse-variance method was used for data pooling, with results expressed as mean differences (MDs) and corresponding 95% confidence intervals (CIs). A *p* value of less than 0.05 was considered indicative of statistical significance. Between-study heterogeneity was evaluated using the I^2^ statistic. In accordance with the Cochrane Handbook, a fixed-effect model was applied when I^2^ was below 50%, whereas a random-effects model was used when heterogeneity exceeded this threshold.

All analyses were conducted across different follow-up time points. To further examine the temporal effect of platelet-rich plasma (PRP), meta-regression was performed using follow-up duration as a covariate. Assessment of publication bias was not undertaken due to the limited number of studies available at each time point.

For additional clinical and radiographic outcomes, only qualitative synthesis was performed, and findings were summarized in tabular form, given the small number of studies and substantial heterogeneity in outcome measurement methods.

## 3. Results

### 3.1. Literature Selection

**Figure 1:**
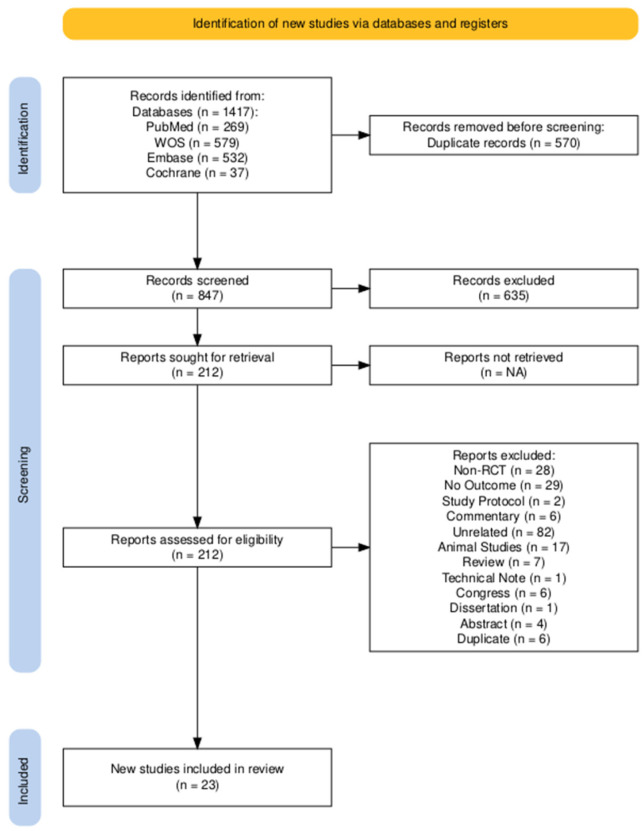
Flow chart of literature search and screening.

A comprehensive search yielded a total of 1416 articles from all databases. Following the removal of 569 duplicate entries, 847 articles were screened based on specific inclusion and exclusion criteria. Upon reviewing titles and abstracts, 212 articles were shortlisted for further evaluation. After a detailed full-text review, 188 articles were subsequently excluded due to various reasons such as no RCT (*n* = 28), no outcomes (*n* = 28), study protocol (*n* = 2), commentary (*n* = 6), unrelated (*n* = 82), animal studies (*n* = 17), review (*n* = 7), technical note (*n* = 1), congress (*n* = 6), dissertation (*n* = 1), abstract (*n* = 4) and duplicate publication (*n* = 6). Ultimately, 23 articles were deemed eligible for inclusion in this study, with 2 articles emanating from a singular RCT and 4 articles emanating from another singular RCT, all reporting different outcomes. The specific outcomes and various sample sizes were extracted and reported accordingly. To avoid double-counting participants for any given outcome or time point, we follow the MOoR Framework, assessing four components: eligibility criteria, data extraction, risk of bias, and synthesis and presentation of findings. Figure 1 shows the flow chart of systematic literature search and screening.

### 3.2. Basic Characteristics of the Literature

The 23 included articles [5,32,33,34,35,36,37,38,39,40,41,42,43,44,45,46,47,48,49,50,51,52,53] involved 19 RCTs containing 1072 patients. Among these RCTs, one RCT published four articles [41,42,50,51] and another RCT published two articles [44,45], all of which were included because they reported different outcomes. Of these 1072 patients, 547 patients (51.0%) were assigned in the PRP group, while 525 (49.0%) patients in the non-PRP group. In total, 72 patients were lost to the final follow-up. All of the included trials were two-arm studies except for one four-arm study. The included studies were published in English-language journals between 2005 and 2024. Sample sizes ranged from 20 to 120 participants, with mean patient ages varying between 26.1 and 37.2 years. The mean duration of follow-up ranged from 3 to 24 months. The percentages of male patients were reported in 22 studies, ranging from 44% to 100%.

Regarding the graft sources selected for ACLR, only one of the 19 RCTs used allograft patellar tendon grafts, and the remaining studies used autograft grafts. For the classification of autograft graft types, five RCTs used bone-patellar tendon-bone grafts and fourteen used hamstring tendon grafts. The internal fixation methods in the femoral and tibial tunnels and detailed rehabilitation protocol were reported in 20 and 15 studies, respectively. Femoral side mainly used cross-pin and EndoButton device, while tibial side mainly used interference screw. The basic characteristics of the included studies and the surgical details are shown in Table 1 and Table 2, respectively.

Table 3 summarizes the preparation methods and application protocols of platelet-rich plasma (PRP) across the included studies. In 14 randomized controlled trials (RCTs), PRP was applied intraoperatively to the femoral tunnel, tibial tunnel, graft substance, and intra-articular space, either alone or in various combinations, whereas four RCTs were applied PRP to the harvest site of the bone–patellar tendon–bone (BPTB) autograft, and only one study reported postoperative intra-articular injection. The volume of PRP administered varied widely, ranging from 3 mL to 40 mL, while the volume of whole blood collected for preparation ranged from 10 mL to 450 mL, both reported in 15 RCTs. Anticoagulation protocols were described in 13 RCTs, most commonly using citric acid or citrate-based agents. A variety of preparation systems were utilized, with a double-spin centrifugation method reported in three RCTs. PRP was administered in different formulations, including liquid PRP in six RCTs and activated gel-form PRP (using thrombin or calcium chloride) in eight RCTs, while four studies used both liquid and gel formulations.

### 3.3. Quality Assessment

The results of the risk-of-bias assessment are summarized in Table 4. Among the included randomized controlled trials (RCTs), an overall moderate to high risk of bias was observed, with only three studies demonstrating low risk across all evaluated domains. The most common sources of bias were related to inadequate allocation concealment and lack of blinding.

Clinical outcomes

### 3.4. VAS Score

**Figure 2:**
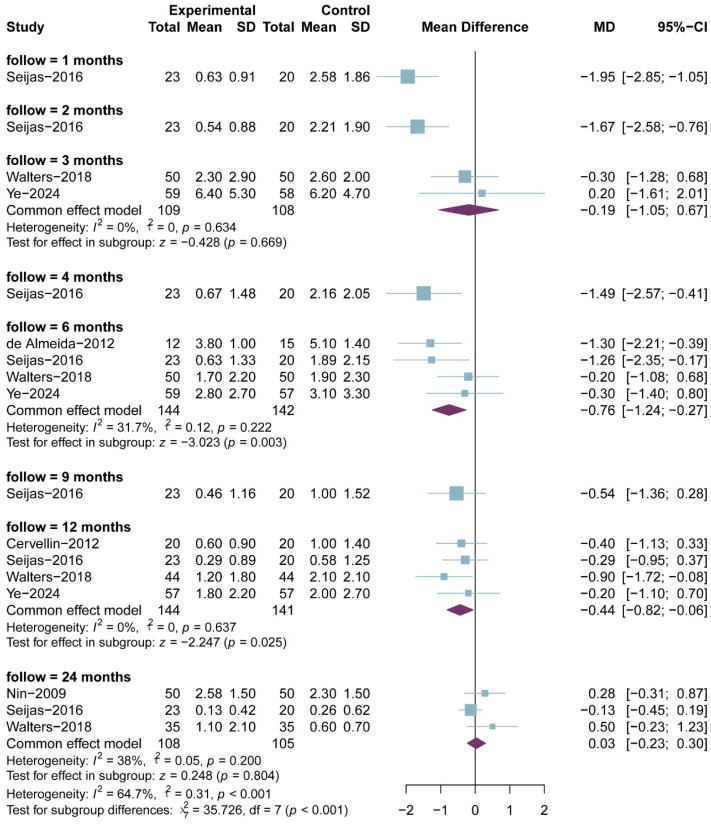
Meta-analysis of VAS score.

**Figure 3:**
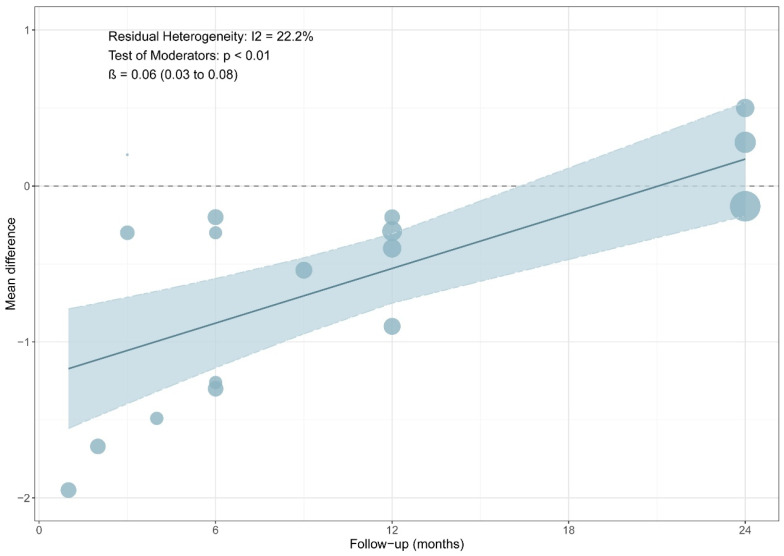
Meta-regression of VAS score.

**Figure 4:**
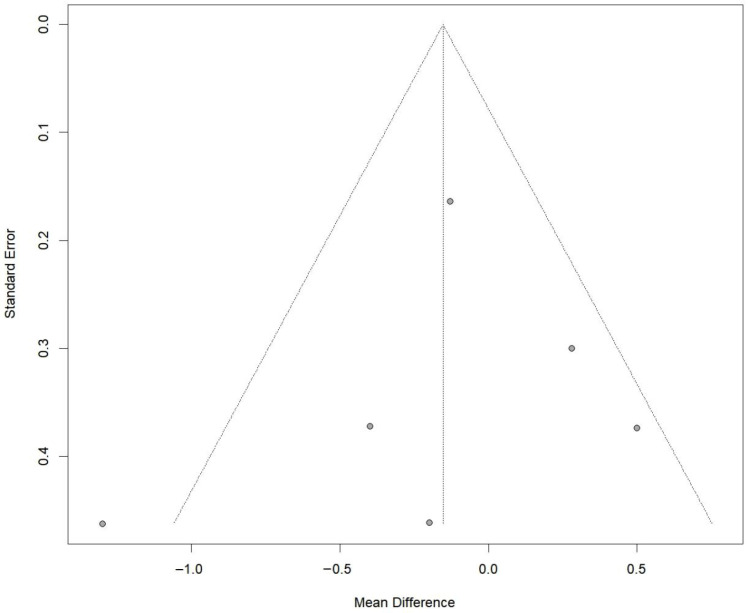
Funnel plot analysis illustrating the publication bias for VAS.

**Figure 5:**
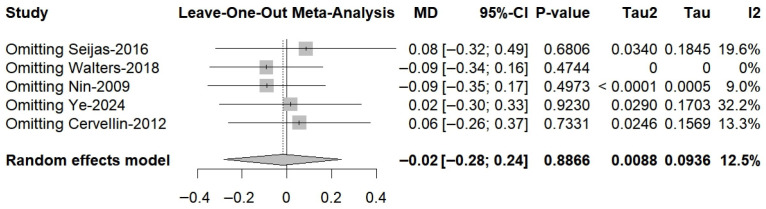
Sensitivity analyses for VAS score.

A total of six RCTs comprising 197 in the PRP group and 197 patients in the non-PRP group, reported VAS scores; the results of the 1-, 2-, 3-, 4-, 6-, 9-, 12-, and 24-month follow-up were reported in one, one, two, one, four, one, four and three RCTs, respectively. All included studies utilized a 0–10 scale for pain assessment. The results of the meta-analysis showed that there were significant differences in the postoperative VAS scores between the two groups at 6-month follow-up (MD, −0.76; 95% CI, −1.24 to −0.27; *p* = 0.003; I^2^ = 31.7%) and 12-month follow-up (MD, −0.44; 95% CI, −0.82 to −0.06; *p* = 0.025; I^2^ = 0.0%) (Figure 2). Although the results of 1-, 2-, and 4-month follow-up were statistically different, only one study was included and it was from the same RCT. Meta-regression showed that the two group differences in VAS scores changed significantly with follow-up time (ß = 0.06 (0.03 to 0.08); residual heterogeneity: I^2^ = 22.2%; test of moderators: *p* < 0.01) (Figure 3). The heterogeneity was 65.9% before meta-regression, and reduced by 84.8% to 22.2% after meta-regression, indicating that follow-up time explained most of the heterogeneity, and the results after regression could be basically considered as homogeneous. Also, we performed sensitivity analysis and publication bias for VAS score. The scatter point of de Almeida et al. [33] study was distributed outside the range of confidence interval (Figure 4), while the sensitivity analysis also proved that after exclusion of the study of de Almeida et al. [33], there was significant decrease in the overall heterogeneity (I^2^ = 12.5%), suggesting a possible source of bias (Figure 5). This may be likely due to the relatively small total sample sizes and short follow-up time.

### 3.5. IKDC Score

**Figure 6:**
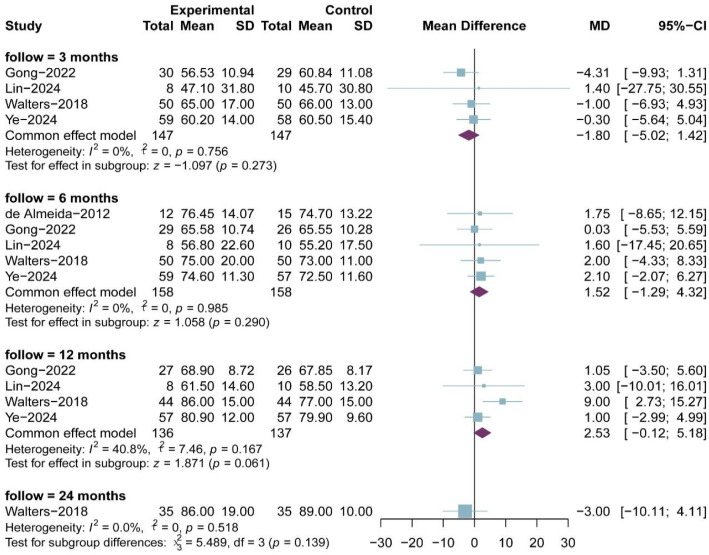
Meta-analysis of IKDC score.

Five RCTs comprising 139 and 143 patients in the PRP and non-RPR groups, respectively, reported IKDC scores; the results of the 3-, 6-, 12-and 24-month follow-up were reported in four, five, four and one RCTs, respectively. All included studies utilized a 0–100 scale IKDC subjective score. The meta-analysis showed that there was no significant difference in IKDC scores between the two groups at 3-month follow-up (MD, −1.80; 95% CI, −5.02 to 1.42; *p* = 0.273; I^2^ = 0.0%), 6-month follow-up (MD, 1.52; 95% CI, −1.29 to 4.32; *p* = 0.290; I^2^ = 0.0%) and 12-month follow-up (MD, 2.53; 95% CI, −0.12 to 5.18; *p* = 0.061; I^2^ = 40.8%) (Figure 6). Only one study reported results at 24 months with no statistical difference. The result of meta-regression was not statistically different (ß = 0.08 (−0.23 to 0.40); residual heterogeneity: I^2^ = 2.2%; test of moderators: *p* = 0.608).

### 3.6. Lysholm Score

**Figure 7:**
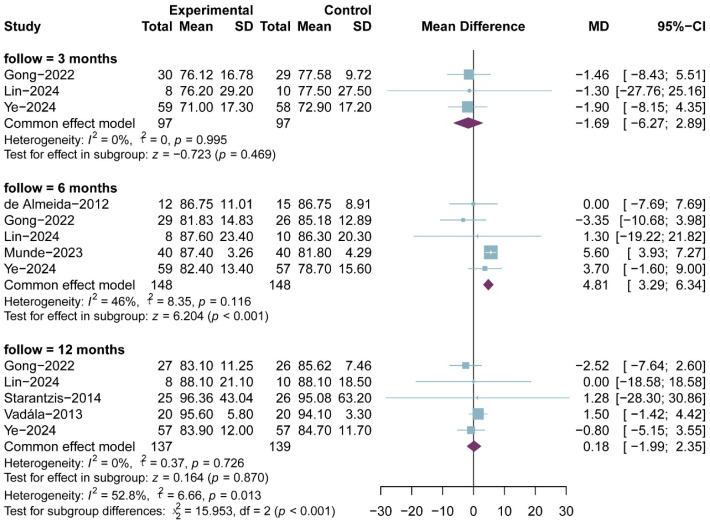
Meta-analysis of Lysholm score.

**Figure 8:**
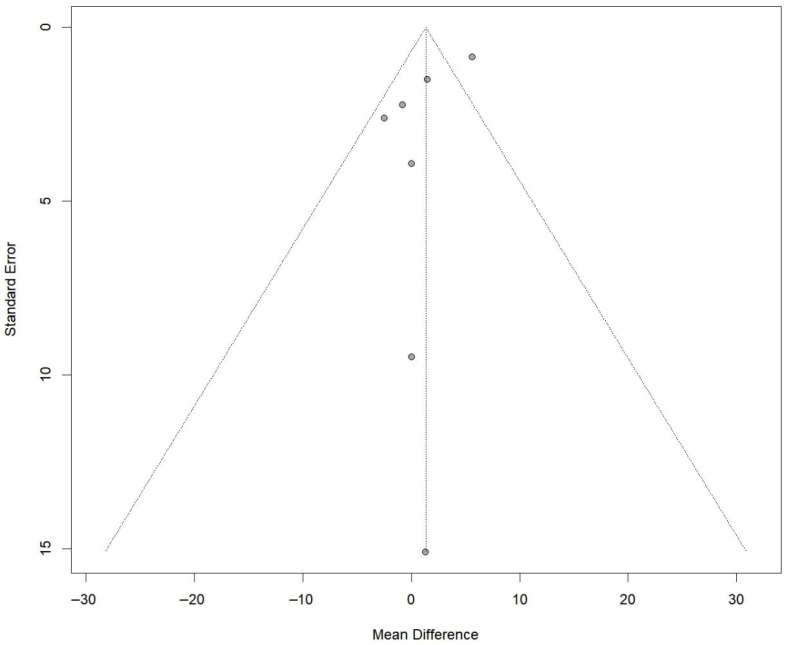
Funnel plot analysis illustrating the publication bias for Lysholm score.

**Figure 9:**
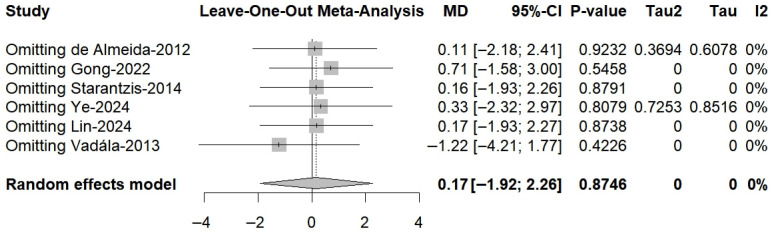
Sensitivity analyses for Lysholm score.

Lysholm scores were reported in seven RCTs, comprising 189 in the PRP group and 194 patients in the non-RPR group; the results of the 3-, 6- and 12-month follow-up were reported in three, five and five RCTs, respectively. All included studies used a 0–100 scale Lysholm scores. The meta-analysis showed that there was no significant difference in Lysholm scores between the two groups at 3-month follow-up (MD, −1.69; 95% CI, −6.27 to 2.89; *p* = 0.469; I^2^ = 0.0%) and 12-month follow-up (MD,0.18; 95% CI, −1.99 to 2.35; *p* = 0.870; I^2^ = 0.0%), but with a significant difference at 6-month follow-up (MD, 4.81; 95% CI, 3.29 to 6.34; *p* < 0.001; I^2^ = 46.0%) (Figure 7). The result of meta-regression was not statistically different (ß = −0.11 (−0.79 to 0.56); residual heterogeneity of the revisions can be seen in page (8)-Lines 389–397 and page (10)-Lines 470–483 of the revised manuscript. ty: I^2^ = 47.8%; test of moderators: *p* = 0.743). We conducted sensitivity analysis and publication bias for Lysholm score. The scatter point of Munde et al., 2023 [38], appeared outside the range of confidence interval (Figure 8), while the sensitivity analysis also proved that after exclusion of that study, the overall heterogeneity dropped substantially (I^2^ = 0.0%) (Figure 9), suggesting a possible source of bias.

### 3.7. Tegner Score

**Figure 10:**
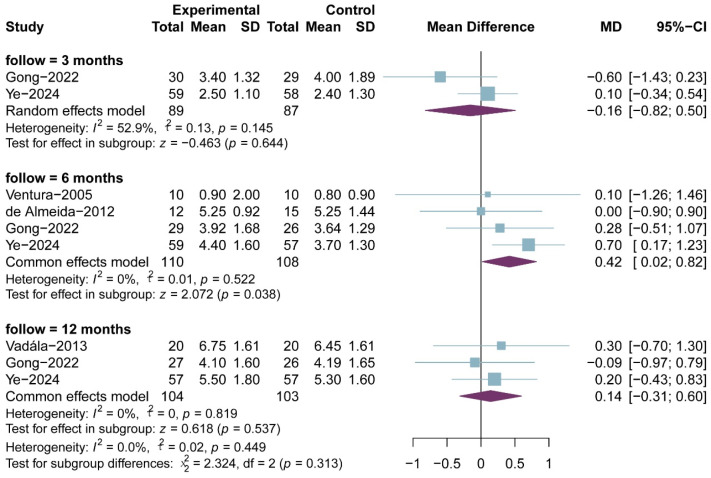
Meta-analysis of Tegner score.

Tegner scores were reported in five RCTs, comprising 126 in the PRP group and 128 patients in the non-RPR group; the results of the 3-, 6- and 12-month follow-up were reported in two, four and three RCTs, respectively. All included studies used a 0–10 scale Tegner scores. The meta-analysis showed that there was no significant difference in Tegner scores between the two groups at 3-month follow-up (MD, −0.12; 95% CI, −0.82 to 0.50; *p* = 0.644; I^2^ = 52.9%) and 12-month follow-up (MD, 0.14; 95% CI, −0.31 to 0.60; *p* = 0.537; I^2^ = 0.0%), but with a significant difference at 6-month follow-up (MD, 0.42; 95% CI, 0.02 to 0.82; *p* = 0.038; I^2^ = 40.8%) (Figure 10). The result of meta-regression was not statistically different (ß = 0.02 (−0.06 to 0.09); residual heterogeneity: I^2^ = 22.2%; test of moderators: *p* = 0.680).

### 3.8. Anterior Knee Laxity

Eight studies measured the knee anterior laxity. KT-1000 arthrometer, KT-2000 arthrometer and Rolimeter were used in five, two and one studies, respectively. At 3-month follow-up, two of three studies reported that PRP significantly improved knee stability. At 6-month follow-up, one of three studies reported that PRP significantly improved knee stability. However, no significant differences were shown in four studies reporting follow-up results at 12-month follow-up and in one study reporting follow-up results at 24-month follow-up. The results are provided in Table S2.

### 3.9. Physical Examinations and Other Outcomes

The results of the ACL physical examinations are shown in Table 5. One RCT showed PRP was superior to non-PRP in Anterior drawer test, Lachman test and Pivot shift test at 3-month follow-up, but there was no statistical difference in the other results. The results of KOOS score, Victorian Institute Sport Assessment Scale, Kujala score, isokinetic strength and proprioception are provided in the Table S3.

### 3.10. Radiological Outcomes

Imaging-based assessment of the treatment effects of platelet-rich plasma (PRP) was reported in 21 studies, utilizing magnetic resonance imaging (MRI) in 16 studies, computed tomography (CT) in 4 studies, ultrasound in 1 study, and X-ray in 1 study. Overall, MRI was the most frequently used modality and was primarily employed to evaluate signal changes in the bone tunnels, the intra-articular portion of the graft, and defects at the bone–patellar tendon–bone (BPTB) harvest site, thereby assessing bone tunnel healing, graft maturation, and donor site recovery. MRI was also used to evaluate femoral and tibial tunnel widening. CT imaging was mainly utilized to measure the diameters of femoral and tibial tunnels and to assess graft maturation. Ultrasound was used for assessing harvest site healing and X-ray was used for assessing widening of tibial tunnel. In many of the included studies, multiple assessments were used for the same indicator, so in the descriptive analysis below, we present results based on the number of assessments rather than the number of studies.

For graft maturation and integration, significant improvements were reported in 6 of 33 assessments (18.1%) at 1~4-month follow-up, 10 of 21 assessments (47.6%) at 6-month follow-up and 0 of 12 assessments (0.0%) at 12-month follow-up for the PRP group. None of the studies reported statistically different results for tunnel widening. One RCT showed PRP was superior to non-PRP in harvest site healing at 4-month follow-up and another RCT showed PRP was superior to non-PRP at 6-month follow-up, but there was no statistical difference in the other assessments. The main outcomes and the significant findings in each primary study are shown in Table S4.

### 3.11. Data Synthesis of PRP Parameters

Out of the 23 articles, 52% utilized gel PRP, 26% utilized liquid PRP, 17% used both gel and liquid, while 4% of the studies did not report the form of PRP used. In terms of the type of leukocyte, 39% of the studies used leuskocye-rich, 22% used leukocyte-poor, and 39% did not report. In total, 9% of the studies applied the PRP inside the tunnels, 65% of them applied the PRP between the strands of the graft and inside the tunnels, and 4% used intra-articular injection. In terms of the time of PRP application, 87% of the studies used intra-operatively, 4% used postoperatively, and 9% of the studies provided no data on that. The synthesis of PRP parameters is presented in Table 5.

## 4. Discussion

This meta-analysis aimed to critically evaluate the role or effect of platelet-rich plasma (PRP) in ACL reconstruction in terms of clinical and radiological outcomes. The main significant finding of this meta-analysis was that PRP application in ACL reconstruction can produce short-term (6 months) but not long-term clinical outcomes, including VAS score, Lysholm score and Tegner score. There was a significant difference between PRP group and non-PRP group in terms of VAS score at 6- and 12-month follow-ups. That means the patients who received PRP had short to medium pain relief. There were no significant differences in subjective IKDC score, Lysholm (at 3 and 12 months), Tegner score (at 3 and 12 months) between the two groups. Upon comparing other secondary outcomes such as anterior knee laxity, KOOS score, Objective IKDC score, Kujala score, proprioception, and isokinetic strength, no remarkable differences were observed in different follow-ups. In terms of radiological outcomes, significant improvement on graft maturation and integration were mainly reported at 6-month follow-up (47.6%) for the PRP group. When considering graft preparation methods, Pavan et al. [54] found that different tripled tendon graft configurations exhibit similar biomechanical properties, indicating that intrinsic tendon characteristics may play a larger role than the grafting technique itself in influencing functional outcomes. Similarly, Rovere et al. [55] highlighted that using a quadrupled semitendinosus graft can achieve a thicker graft diameter while reducing donor site morbidity, which may impact both early rehabilitation and graft survival.

With the proliferation of literature on the use of PRP in ACL reconstruction, many studies on different PRP type, form and preparation have been published. These studies predominantly summarize and critically evaluate the role of PRP in terms of clinical, functional and radiological outcomes. Several systematic reviews and meta-analyses have been conducted on PRP application in ACL reconstruction and they have unveiled and revealed satisfactory results. Davey et al. [29] reported no significant differences in Tegner score, Lysholm score and IKDC score in 7 studies evaluating PRP versus control in ACL reconstruction. However, our meta-analysis demonstrated significant differences in Tegner score (6 months) and Lysholm score (6 months), but no long-term benefit (postoperative 12 months). The meta-analysis of Davey et al. [29] revealed no significant difference between the PRP and the control groups at a mean follow-up time of 7.5 months (range: 6–23 months) in terms of VAS score (MD, −0.40; 95% CI, −0.98 to 1.0.18, I^2^ = 78%, *p* = 0.18). Contrastingly, the results of the VAS score of our meta-analysis showed significant difference between the PRP group and non-PRP group at 6 months (MD, −0.76; 95% CI, −1.24~−0.27, I^2^ = 31.7%, *p* = 0.003) and 12 months (MD, −0.44; 95% CI, −0.82 to −0.06, I^2^ = 0%, *p* = 0.025), respectively. But there was no difference after 24 months follow-up (MD, 0.03; 95% CI, −0.23 to 0.30, I^2^ = 38%, *p* = 0.804). There was a significant heterogeneity: I^2^ = 64.7% after the meta-analysis. To confirm the influence of time on the PRP-related reduction in pain, we conducted a univariate regression analysis of the VAS score, and the heterogeneity (I^2^) was reduced to 22.2%.

A meta regression analysis was performed using follow-up duration as a covariate, as this variable was consistently reported across studies and demonstrated a significant contribution to heterogeneity. However, other potentially important effect modifiers, including PRP formulation such as leukocyte-rich versus leukocyte-poor, application site, activation method, and graft type, could not be formally examined due to inconsistent reporting and the limited number of studies available per subgroup. Consequently, residual confounding from unmodeled between study differences cannot be excluded. Future well-designed trials with standardized reporting of PRP characteristics and surgical parameters are warranted to better clarify these sources of heterogeneity.

De Andrade et al. [28] reported no statistically significance differences in PRP group compared to non-PRP group in terms of graft ligamentization (SMD, 0.01; 95% CI −0.37 to 0.39), tunnel widening (SMD, 0.71; 95% CI, −0.12 to 1.54), knee laxity, IKDC score, and Tegner score, but demonstrated significant differences in VAS score (SMD, −0.29; 95% CI, −0.84 to 0.25, *p* < 0.01) and Lysholm score (SMD, 5.38; 95% CI, 2.16 to 8.60, *p* < 0.01). They suggest that although there were significant differences in VAS score and Lysholm score, the results were too small to lead to a significant clinical effect. Our meta-analysis revealed similar outcomes except for VAS score, Lysholm score (6 months) and Tegner score (6 months). Lower VAS score in patients treated with PRP compared with controls after ACL reconstruction in our study is consistent with the outcome of the study conducted by de Andrade et al. [56] at 6–12-month follow-ups. Additionally, in the meta-analysis conducted by Zheng-Tao et al. [57] to assess the efficacy of PRP on pain relief, functional improvement, and radiological changes between PRP and control groups, they found no statistically significant difference in VAS (MD, −0.47, *p* = 0.04), subjective IKDC score (MD, 3.99, *p* = 0.03), Lysholm score (MD, 2.30, *p* = 0.32), objective IKDC score (RR,1.03, *p* = 0.09) and knee joint laxity (MD: 0.17, *p* = 0.28) at 12-month follow-up. Contrastingly, they found significant improvement with regard to VAS score (MD, −1.12; 95% CI, −1.92 to −0.31, *p* = 0.007), subjective IKDC score (MD, 6.08; 95% CI, 4.39 to 7.77, *p* < 0.00001) and Lysholm score (MD, 8.49; 95% CI, 1.63 to 15.36, *p* = 0.02) at 6-month follow-up. They concluded that application of PRP in ACL reconstruction could provide short-term (6 months) but not long-term clinically important pain reduction. Our meta-analysis found short-term (6 months) to moderate-term (12 months) pain reduction in the PRP group compared to the non-PRP group, but no long-term benefit (24 months).

However, it is important to recognize that statistical significance does not necessarily equate to clinical relevance. One of the statistically significant findings was the higher Lysholm score observed in the PRP group at 6 months (MD 4.81). Given that the minimal clinically important difference (MCID) for the Lysholm score has been reported to be approximately 10 [58], this improvement is unlikely to represent a clinically meaningful benefit. Similarly, the PRP group demonstrated lower visual analog scale (VAS) pain scores at both 6 months (MD −0.76) and 12 months (MD −0.44). However, considering the subjective nature of VAS and its reported MCID range of 1.5–3.0 [19], these differences are small and unlikely to be perceived as clinically significant by patients [59]. Overall, while PRP may confer modest biological effects in the short term, these changes are unlikely to translate into meaningful improvements in patient-reported pain or functional outcomes. Therefore, clinicians should interpret these findings with caution and incorporate shared decision-making when discussing the potential role of PRP with patients, taking into account individual expectations and treatment goals.

Regarding radiological changes such as healing and vascularization of the graft, tunnel widening and donor site healing, they stated that not every single study reported advantageous effect of PRP compared with control, PRP treatment favored slightly desirable effect than control (*p* > 0.05). Cao Yi and Ye-da Wan performed a systematic review of randomized controlled trials aimed to identify the effectiveness of PRP in ACL reconstruction [27]. They qualitatively synthesized clinical and imaging outcomes such as bone tunnel healing, graft remodeling, donor site healing, tunnel widening, IKDC score, Lysholm score, Tegner score, knee anteroposterior and rotational laxity, range of motion and VAS. They found significant improvement on graft remodeling (one of five studies, 20.0%), bone tunnel healing (three of five studies, 60.0%), donor site healing (two of four studies, 50.0%) and bone tunnel widening (one of five studies, 20.0%) for PRP group. PRP application did not improve in terms of the aforementioned clinical outcomes. Growth factors such as platelet-derived growth factor (PDGF) represent one of the most important molecular families involved in healing, which helps in tissue remodeling [60]. Zhu Ting et al. found that PRP application could reduce postoperative pain (VAS) and improve knee function (IKDC score, Lysholm score, Tegner score, KT-1000, and pivot-shift test), in the short term (before 6 months). Our study supports their VAS outcome, but our results extend postoperative pain reduction benefit to 12 months due to newly included studies and a large sample size. They attributed the short and medium effects of PRP on VAS score to the biological properties of PRP [33,61,62]. They also concluded that PRP does not seem to be an effective treatment for improvement of clinical outcomes, and suggested future research to be conducted. McRobb Jonathon et al. [63] conducted a systematic review of the effect of PRP on pain, knee stability and, resultant knee function, but could not arrive at a consensus. Regarding the VAS score, they identified a research gap that future research should elaborate on the impact of PRP on pain since the synthesis of the available studies failed to reach statistical significance. In the aspect of healing, they reported that PRP seems to have an early influence on healing based on the available studies. De Mos et al. [64] suggested in their in vivo study that PRP application could lead to accelerated remodeling and angiogenesis, and may promote repair of injured tendon. A recent scoping review has highlighted the impact of different forms of PRP in ACL reconstruction. Their finding showed that there is no vivid indication of PRP in clinical practice due to scattered evidence of PRP augmentation in ACL reconstruction [65]. Our meta-analysis of four available studies showed that PRP can reduce postoperative pain in short to medium term, but no long-term postoperative pain relief. PRP also exhibited short term benefits in Lysholm score and Tegner score, but no long-term benefit. In terms of radiological findings, about half of the assessments favored PRP to facilitate the graft maturation and integration at 6-month follow-up. The results of other clinical and radiological outcomes were not significant.

The strengths of this study are noteworthy. First, a rigorous and comprehensive search strategy, including supplementary manual screening, was employed to ensure that the synthesized evidence was as complete and reliable as possible. Second, only randomized controlled trials were included, and the baseline characteristics across studies were generally comparable, enhancing the internal validity of the analysis. Third, multiple clinical outcomes were evaluated, allowing for a comprehensive comparison between the PRP and non-PRP groups across a range of relevant domains.

Certainly, our study may also have some shortcomings: (1) there were only a small number of long-term follow-up studies; (2) fewer quantitative outcomes made it difficult for us to run an analysis for certain studies; and (3) although short-term benefits of PRP were observed, these findings should be interpreted with caution, as only a minority of included trials were judged to be at low risk of bias across all domains. Common methodological concerns included allocation concealment and blinding procedures, which may have influenced subjective outcomes such as pain scores. Sensitivity analyses restricted to lower risk studies yielded generally consistent results, although reduced precision was observed. Additionally, publication bias was detected based on funnel plot assessment. Nevertheless, the overall certainty of evidence remains moderate due to methodological limitations. Therefore, future well-designed, adequately powered trials are required to clarify the true clinical value of Platelet-Rich Plasma Injection in Anterior Cruciate Ligament Reconstruction.

Finally, it is worth noting that emerging technologies may help optimize outcomes tracking and clinical decision-making. Rovere et al. [66] highlighted that blockchain combined with AI could improve orthopedic registry management, data security, and patient-specific analysis, which may facilitate future evaluations of PRP or surgical techniques in ACL reconstruction.

## 5. Conclusions

The application of PRP in ACL reconstruction may produce short-term but not long-term clinical outcomes, including VAS score, Lysholm score and Tegner score. No remarkable differences were noted in other clinical and radiological outcomes such as Anterior knee laxity, subjective and objective IKDC score, KOOS Score, Kujala score, Proprioception, Isokinetic strength, graft maturation and integration, tunnel widening, and healing. While some short-term statistical differences exist, their magnitude and durability do not yet justify routine clinical adoption of PRP in ACL reconstruction. Larger-sample higher-quality studies with more quantitative outcomes are needed to support our results and further explore the comprehensive comparison between different forms of PRP.

## Figures and Tables

**Table 1:** Characteristics of the included studies.

First Author-Year	Study Design-LOE	Sample Size		Age (Years)		Sex (Male /Female)		Follow-Up (Months)	Drop Out	
		PRP	No PRP	PRP	No PRP	PRP	No PRP		PRP	No PRP
Ventura-2005	RCT-I	10	10	36.6 ± 9.3	30.2 ± 5.3	9/1	9/1	6	0	0
Orrego-2008	RCT-II	29	29	30 (15–57)		85%		6	3	2
Nin-2009	RCT-I	50	50	26.1 (14–57)	26.6 (15–59)	40/10	38/12	6	0	0
Silva-2009	RCT-II	30	10	26.8 ± 5.3		38/2		3	0	0
Vogrin-2010	RCT-I	25	25	37.2 ± 8.4	32.6 ± 12.3	15/10	16/9	3	4	5
Vogrin-2010	RCT-I	25	25	35.4 ± 10.0	33.0 ± 12.5	15/10	16/9	6	3	2
Cervellin-2012	RCT-I	20	20	22.9 ± 4.3	22.7 ± 3.5	20/0	20/0	12	2	3
de Almeida-2012	RCT-I	12	15	25.8 (18–44)	23.1 (15–34)	10/2	14/1	6	2	2
Mirzatolooei-2013	RCT-I	25	25	26.4 (18–40)	26.9 (18–40)	20/3	22/1	3	4	5
Rupreht-2013	RCT-I	25	25	37.2 ± 8.4	32.6 ± 12.3	15/10	16/9	6	4	5
Rupreht-2013	RCT-I	25	25	37.2 ± 8.4	32.6 ± 12.3	15/10	16/9	6	2	2
Seijas-2013	RCT-I	50	50	NA	NA	NA	NA	12	1	1
Vadalà-2013	RCT-II	20	20	34.5 (18–48)		20/0	20/0	14.7	0	0
Starantzis-2014	RCT-I	30	30	29.4 ± 7.3	31.3 ± 8.0	38/13		12	5	4
Seijas-2015	RCT-I	23	21	NA	NA	20/3	17/3	12	0	1
Seijas-2016	RCT-I	23	21	NA	NA	20/3	17/3	24	0	1
Walters-2018	RCT-II	30	29	30 ± 12		10/17	12/11	24	7	8
Mahdi-2019	RCT-I	14	13	25.77	25.77	14/0	13/0	3	0	0
Gong-2022	RCT-I	30	30	33.5 ± 8.97	34.9 ± 9.68	18/12	21/9	12	3	4
Kumar-2022	RCT-I	35	35	28.34 ± 4.32	29.71 ± 2.99	22/13	23/12	3	0	0
Munde-2023	RCT-I	44	43	28.37 ± 2.8	27.4 ± 7.0	36/4	38/2	6	4	3
Lin-2024	RCT-I	10	10	28.4 ± 7.8	29.7 ± 9.9	6/2	6/4	12	2	0
Ye-2024	RCT-I	60	60	28.0 ± 7.9	30.0 ± 8.0	43/17	41/19	12	3	3

Ages are reported as mean ± SD (range). LOE, level of evidence; RCT, randomized controlled trial; NA, not available; PRP, platelet-rich plasma.

**Table 2:** Surgical Parameters.

Author-Year	Graft Source	Graft Type	Fixation Method	Rehabilitation Protocol
Ventura-2005	Autograft	Quadrupled hamstring tendon	Transcondylic fixation (femoral side) and interference screw (tibial side)	Immediate post-operative mobilization without a knee brace, protected weight bearing for 3 weeks, and return to sporting activities at 6 months.
Orrego-2008	Autograft	Quadrupled hamstring tendon	Biodegradable transfixing pin (femoral side) and biodegradable interference screw (tibial side)	NA
Nin-2009	Allograft	Patellar tendon	2 biodegradable cross pins (femoral side) and biodegradable interference screw (tibial side)	Immediate post-operative immobilized with a knee brace, whole range of movement after 10 days, Cycling at 2 to 3 months, straight-line running, at 4 months, and sports at 6 months.
Silva-2009	Autograft	Double-bundle hamstring tendon	EndoButton CL devices (femoral side) and bioabsorbable interference screw (tibial side)	Immobilization of the knee with a brace in full extension for a week after surgery, after which mobilization is started, below 90 of flexion until the fourth week and then, increasing the flexion of the knee 15 every week. Protection from weight bearing is used for 5 weeks after surgery.
Vogrin-2010	Autograft	Double-looped hamstring tendon	2 bioabsorbable cross pins (femoral side) and bioabsorbable interference screw (tibial side)	No rehabilitation brace was used postoperatively. Running at 12 weeks and contact sports at 6 months provided that the patient had no knee joint effusion, achieved a full range of motion and obtained muscle strength of at least 90% compared to the contralateral leg.
Vogrin-2010	Autograft	Double-looped hamstring tendon	2 bioabsorbable cross pins (femoral side) and bioabsorbable interference screw (tibial side)	No rehabilitation brace was used postoperatively. Running was allowed at 12 weeks and contact sports at 6 months in cases with no knee-joint effusion, full range of motion and obtained muscle strength of 90% compared with the contralateral leg.
Cervellin-2012	Autograft	Bone-patellar tendon-bone	NA	Weight bearing with the knee extended for the first 15 days, followed by partial weight bearing for the next 15 days. walking without the aid of crutches during the second months. During the third and fourth months, closed kinetic chain exercises and gradual return to sports activity were allowed.
de Almeida-2012	Autograft	Bone-patellar tendon-bone	A transverse double pin absorbable system (femoral side) and an absorbable interference screw (tibial side).	Early range of motion and progressive weight-bearing with crutches for 3 weeks.
Mirzatolooei-2013	Autograft	Quadrupled hamstring tendon	A cross-pin (femoral side) and bio-absorbable interference screw (tibial side)	Wore a knee immobilizer in full extension for two weeks.
Rupreht-2013	Autograft	Double-looped hamstring tendon	2 bioabsorbable cross pins (femoral side) and one bioabsorbable interference screw (tibial side)	NA
Rupreht-2013	Autograft	Double-looped hamstring tendon	2 bioabsorbable cross pins (femoral side) and one bioabsorbable interference screw (tibial side)	NA
Seijas-2013	Autograft	Bone-patellar tendon-bone	Hydroxylapatite screws (both femoral and tibia sides)	Postoperatively, the knee was immobilized with 2 plaster splints. At week 4, progressive weight-bearing ambulation was allowed as pain tolerated. Pool exercises were started at week 6, outdoor cycling at month 3, progressive running at month 4, and return to unrestricted sporting activities at month 6.
Vadalà-2013	Autograft	Hamstring tendon	Swing-Bridge device (femoral side) and the Evolgate (tibial side)	Post-operatively, all patients started weight-bearing with the use of crutches the day after the operation. Within the first 6 weeks, patients started progressive isotonic and isokinetic exercises. Patients involved in sports activities were allowed to return to practice their sport 6 months after surgery, and patients involved in noncontact sports after 4 months.
Starantzis-2014	Autograft	Quadrupled hamstring tendon	Crosspin or Endobutton (femoral side) and biodegradable interference screw + bone bridge suture anchoring (tibial side)	NA
Seijas-2015	Autograft	Bone-patellar tendon-bone	NA	NA
Seijas-2016	Autograft	Bone-patellar tendon-bone	NA	NA
Walters-2018	Autograft	Bone-patellar tendon-bone	Two titanium cannulated interference screws (both femoral and tibial side)	NA
Mahdi-2019	Autograft	Quadrupled-strand hamstring tendon	“Suspensory” fixation mechanism (Endobutton) in both sides.	NA
Gong-2022	Autograft	Quadrupled-strand hamstring tendon	Endobutton (femoral side) and Intrafx Device (tibial side)	Partial weight-bearing was permitted with the brace locked in full extension within 4 weeks after operation. Full weight-bearing with the brace was permitted at the seventh week after operation; full weight-bearing without the brace was permitted at the third month after operation. Walking, running and contact sports were permitted after 3, 6 and 9 months, respectively.
Kumar-2022	Autograft	Hamstring tendon	Endobutton (femoral side) and biodegradable interference screw (tibial side)	Standard ACL rehabilitation protocol which included gaining a range of motion, muscle strength and returning to preinjury status in phasic manner.
Munde-2022	Autograft	Quadrupled hamstring tendon	Adjustable loop button (femoral side) and biodegradable interference screw (tibial side)	In the first phase (up to 2 weeks) all patients were started on full weight-bearing walking with crutches, isometric quadriceps exercises, etc. In the second phase (2–4 weeks), quadricep strengthening exercises, knee ROM exercises, and balance exercises started. In the third and fourth phase (1–6 months), resistance band quadriceps, hamstring strengthening exercises, jogging, controlled single-leg jumping, plyometric exercises, etc. were done.
Lin-2024	Autograft	Fourth or fifth-strand hamstring tendon	Interference screws (femoral side) and Interference screws + cancellous post-screw (tibial side)	Every subject underwent the program at 1, 3, 5, 7, and 9 weeks post-surgery.
Ye-2024	Autograft	Quadrupled-strand hamstring tendon	Cortical buttons (femoral side) and biodegradable interference screw + cortical button with an adjustable loop (tibial side)	Standardized rehabilitation education from a physical therapist through verbal instruction, printed brochures, and online videos before discharge.

NA, Not Available.

**Table 3:** PRP Parameters.

Author-Year	Processing Machine	Whole Blood Volume (mL)	Anticoagulants	Spin Speed (rpm)	Spin Time (min)	Platelet Concentration (mm^3^)	Activation	Form	Leukocyte	Location	Time Point	Volume (mL)
Ventura-2005	GPS Biomet Merck technique (Biomet Inc., Warsaw, IN, USA)	54	Citric acid	3200	12	NA	Autologous thrombin	gel	rich	Femoral and tibial tunnels	Intraoperative	NA
Orrego-2008	Biomet GPS II kit (Biomet, Warsaw, IN, USA)	57	NA	200	15	NA	Autologous thrombin with CaCl_2_	gel + liqiud	rich	Between the strands of the graft and femoral tunnel	Intraoperative	6
Nin-2009	Beckman J-6B, Beckman Coulter Spain, Madrid, Spain	40	Citric acid	1st: 3000 2nd: 1000	1st: 8 2nd: 6	837,000	CaCl_2_	gel	poor	Graft was covered with gel and inside the tibial tunnel	Intraoperative	4
Silva-2009	Mini GPS III Kit (Biomet)	27	Citric acid	3200	15	NA	Autologous thrombin	gel + liqiud	rich	Between the strands of the graft and inside the femoral tunnel	Intraoperative	3
Vogrin-2010	Magellan (Medtronic Biologic Therapeutics and Diagnostics, Minneapolis, MN, USA) autologous platelet separator	50	10% calcium citrate	NA	NA	978 × 10^9^/L (range 552 to 1326 × 10^9^/L)	Autologous thrombin	gel	rich	Between the strands of the graft and inside the femoral and tibial tunnels.	Intraoperative	6
Vogrin-2010	Magellan autologous platelet separator (Medtronic Biologic Therapeutics and Diagnostics, Minneapolis, MN, USA)	50	10% calcium citrate	NA	NA	962 (552–1326) G/L	Autologous thrombin	gel	rich	Between the strands of the graft and inside the femoral and tibial tunnels.	Intraoperative	6
Cervellin-2012	Gravitational Platelet Separation II (GPS) system (Biomet Biologics, Inc., Warsaw, IN, USA)	54	ACD-A	3200	15	NA	Autologous thrombin with CaCl_2_	gel	rich	Patellar and tendon bone plug harvest site	Intraoperative	NA
de Almeida-2012	A Haemonetics MCS1 9000 cell separator with a specific kit for platelet apheresis 995-E (Haemonetics Corp., Braintree, MA, USA)	450	10% citrate	NA	NA	1,185,166 ± 404,472	Autologous thrombin with CaCl_2_	gel	poor	Patellar tendon defect	NA	20–40
Mirzatolooei-2013	Double syringe system (Arthrex, Naples, FL, USA)	10	NA	1500	5	NA	CaCl_2_	liquid	poor	Between the strands of the graft and inside the femoral and tibial tunnels.	Intraoperative	3.5
Rupreht-2013	Magellan autologous platelet separator (Medtronic Biologic Therapeutics and Diagnostics)	50	NA	NA	NA	NA	Autologous thrombin	gel	rich	Between the strands of the graft and inside the femoral and tibial tunnels.	Intraoperative	6
Rupreht-2013	Magellan autologous platelet separator (Medtronic Biologic Therapeutics and Diagnostics)	50	NA	NA	NA	978 × 10^9^/L (range 552 to 1326 × 10^9^/L)	Autologous thrombin	gel	rich	Between the strands of the graft and inside the femoral and tibial tunnels.	Intraoperative	6
Seijas-2013	PRGF technique (BTI Systems Vitoria, Spain)	NA	NA	NA	NA	NA	No activation	liquid	NA	Suprapatellar joint after portal suture	Intraoperative	8
Vadalà-2013	PRP Fast Biotech kit (MyCells PPT-Platelet Preparation Tube)	10	NA	NA	NA	NA	Autologous thrombin with Ca-gluconate	liquid + gel	NA	Between the strands of the graft and inside the femoral and tibial tunnels.	Intraoperative	15
Starantzis-2014	Biomet GPS III kit (Biomet, Warsaw, IN, USA)	55	ACD-A	3200	15	NA	CaCl_2_.	liquid + gel	rich	Between the strands of the graft and inside the femoral tunnel.	Intraoperative	6
Seijas-2015	NA	20cc	NA	NA	NA	NA	NA	NA	NA	Patellar bone gap, tibial bone gap, and the center of harvest gap.	Intraoperative	4
Seijas-2016	NA	34cc	Sodium citrate	1800	8	NA	CaCl_2_	gel	NA	Patellar bone gap, tibial bone gap, and the center of harvest gap.	Intraoperative	4
Walters-2018	A PRP separation kit and centrifuge system (ACP PRP; Arthrex)	10	ACD-A	1500	5	NA	CaCl_2_	gel	poor	Patellar donor site	NA	3–5
Mahdi-2019	Trima Accel Automated Blood Collection system	100–150	NA	NA	NA	5–7 × 10^7^	No activation	liquid	NA	Inside femoral tunnel and intra-articularly	Intraoperative	6
Gong-2022	Platelet Rich Plasma Preparation Kits (WEGO; Beijing, China) and a centrifuge (WEGO; Beijing, China)	36	ACD-A	NA	NA	NA	No activation	liquid	NA	Inside the bone tunnels and graft	Intraoperative	4
Kumar-2022	NA	NA	NA	NA	NA	NA	No activation	liquid	NA	Between the strands of the graft and inside the femoral and tibial tunnels.	Intraoperative	NA
Munde-2023	Remi R8 C fixed angle microcentrifuge	17	ACD-A	1st: 1500 2nd: 2500	1st: 15 2nd: 10	NA	platelet agitator	gel	NA	Inside the femoral tunnel	Intraoperative	3–4
Lin-2024	NA	30	NA	3200	6	NA	NA	gel	NA	At each end of the graft (bone tunnel side) and into the knee joint.	Intraoperative	NA
Ye-2024	commercially used system (Platelet-Rich Plasma Preparation Kit; WEGO Ltd.)	45	ACD-A	NA	1st: 10 2nd: 15	678 ± 173 × 10^9^/L	No activation	liquid	poor	Intra-articular injection	4 weeks, 8 weeks and 3 months postoperatively	5

NA, Not Available.

**Table 4:** Quality assessment of the RCT studies.

Author-Year	Selection Bias		Performance Bias	Detection Bias	Attrition Bias	Reporting Bias	Other Bias
	Random Sequence Generation	Allocation Concealment	Blinding of Participants and Personnel	Blinding of Outcome Assessment	Incomplete Outcome Data	Selective Reporting	
Ventura-2005	high	high	high	high	high	low	high
Orrego-2008	low	unclear	low	low	low	low	low
Nin-2009	low	low	low	low	low	low	unclear
Silva-2009	unclear	unclear	low	unclear	low	low	unclear
Vogrin-2010	low	low	low	low	low	low	unclear
Cervellin-2012	low	low	low	low	low	low	low
de Almeida-2012	low	low	low	low	low	low	unclear
Mirzatolooei-2013	low	low	unclear	unclear	low	low	unclear
Rupreht-2013	unclear	unclear	low	low	low	low	unclear
Rupreht-2013	low	low	low	low	low	low	unclear
Seijas-2013	unclear	unclear	unclear	low	low	low	unclear
Vadalà-2013	unclear	unclear	unclear	low	low	low	unclear
Starantzis-2014	low	unclear	low	low	low	low	low
Seijas-2015	low	low	low	low	low	low	unclear
Seijas-2016	low	low	low	low	low	low	unclear
Walters-2018	low	low	low	low	low	low	low
Mahdi-2019	unclear	unclear	unclear	unclear	low	low	unclear
Gong-2022	low	unclear	unclear	low	low	low	low
Kumar-2022	low	unclear	unclear	unclear	low	low	low
Munde-2023	low	low	unclear	low	low	low	low
Lin-2024	low	unclear	low	low	low	low	low
Ye-2024	low	low	low	low	low	low	low

**Table 5:** Results of physical examinations.

Outcomes	Author-Year	Grade	PRP Group	Non-PRP Group	*p*-Value	Follow-Up
Anterior drawer test	Mahdai-2019	<5/5–10/>10	12/2/0	1/10/2	<0.0001	1 month
	Ventura-2005	negative/positive	9/1	10/0	NS	6 months
	Kumar-2022	1/2/3	33/2/0	31/4/0	0.303	3 months
	Munde-2023	negative/positive	39/1	33/7	NS	6 months
	Ye-2024	0/1/2/3	54/3/0/0	55/2/0/0	0.65	12 months
Lachman test	Mahdai-2019	<5/6–10	12/2	6/7	0.033	1 month
	Ventura-2005	negative/positive	9/1	9/1	NS	6 months
	Munde-2023	negative/positive	39/1	33/7	NS	6 months
	Mirzatolooei-2013	negative/positive	25/0	25/0	NS	3 months
	Vadála-2013	negative/positive	20/0	20/0	NS	14.7 months
	Kumar-2022	1/2/3	33/2/0	31/4/0	0.808	3 months
	Ye-2024	0/1/2/3	54/0/3/0	55/0/0/2	0.68	12 months
Pivot shift test	Starantzis-2014	negative/positive	30/0	30/0	NS	12 months
	Mahdai-2019	absent/pivot glide	12/2	5/8	0.014	1 month
	Vadála-2013	(neg, +1)/(neg, +1)	16/4	18/2	NS	14.7 months
	Ye-2024	0/1/2/3	54/2/1/0	55/0/1/1	0.68	12 months
Objective IKDC	Nin-2009	A/B/C/D	35/15/0/0	35/13/2/0	NS	24.3 months
	Orrego-2008	(A + B)/(C + D)	24/2	26/1	NS	6 months
	Vadála-2013	A/B/C/D	16/4/0/0	16/4/0/0	NS	14.7 months

IKDC, International Knee Documentation Committee; PRP, Platelet-Rich Plasma; NS, Not Significant.

## Data Availability

The original contributions presented in this study are included in the article. Further inquiries can be directed to the corresponding authors.

## Supplementary Materials

The following supporting information can be downloaded at: https://www.mdpi.com/article/10.3390/bioengineering13040455/s1, Supplementary Table S1. Preferred Reporting Items for Systematic reviews and Meta-Analyses (PRISMA) checklist. Supplementary Table S2. Results of Anterior Knee Laxity. Supplementary Table S3. Results of Other Comparable Continuous Outcomes. Supplementary Table S4. Results of Radiological Outcomes.

## Abbreviations

ACL	Anterior Cruciate Ligament
PRP	Platelet-Rich Plasma
PRISMA	Preferred Reporting Items for Systematic Reviews and Meta-Analyses
IKDC	International Knee Documentation Committee
MD	Mean Difference
CI	Confidence Interval
RCTs	Randomized Controlled Trials
KOOS	Knee Injury and Osteoarthritis Outcome Score
VAS	Visual Analogue Scale
