# Supplementary material for: Role of Platelet-Rich Plasma Injection in Anterior Cruciate Ligament Reconstruction: A Meta-Analysis of Randomized Controlled Trials
Source: Bioengineering (Basel). 2026 Apr 13;13(4):455. doi: 10.3390/bioengineering13040455 (PMC13113828; doi:10.3390/bioengineering13040455)
Supplement: Supplementary file 1 [file bioengineering-13-00455-s001.zip › bioengineering-4188222-supplementary.pdf]

bioengineering

MDPI

Article

Role of Platelet-Rich Plasma Injection in Anterior Cruciate Ligament Reconstruction: A Meta-Analysis of Randomized Controlled Trials

Ahmed Abdirahman Ibrahim [1,2,†], Michael Opoku [1,2,†], Abakar Mahamat Abdraman [1,2,†], Mingqing Fang [1,2], Xu Liu [1,2], Abdulraheem Mustapha [3], Yusheng Li [1,4], Wenfeng Xiao [1,4], Kai Zhang [2,5,*] and Shuguang Liu [6,*]

1 Department of Orthopaedics, Xiangya Hospital, Central South University, Changsha 410008, China; 248119013@csu.edu.cn (A.A.I.); michaelopuku@csu.edu.cn (M.O.); 248119012@csu.edu.cn (A.M.A.); 8303200916@csu.edu.cn (M.F.); liyusheng@csu.edu.cn (Y.L.); xiaowenfeng@csu.edu.cn (W.X.)
2 Xiangya School of Medicine, Central South University, Changsha 410083, China
3 Department of Cardiovascular Surgery, The Second Xiangya Hospital of Central South University,
Changsha 410011, China; aamustapha21@csu.edu.cn
4 National Clinical Research Center for Geriatric Disorders, Xiangya Hospital, Central South University, Changsha 410008, China
5 Department of Orthopaedics, The First People's Hospital of Changde City, Central South University, Changda 415000, China
6 Department of Joint Surgery, Honghui Hospital, Xi'an Jiaotong University, Xi'an 710061, China
* Correspondence: zhangkaichangde@163.com (K.Z.); orth_shuguang@163.com (S.L.)
† These authors have contributed equally to this work.

Supplementary file

Supplementary Table S1. Preferred Reporting Items for Systematic reviews and Meta-Analyses (PRISMA) checklist.

Supplementary Table S2. Results of Anterior Knee Laxity.

Supplementary Table S3. Results of Other Comparable Continuous Outcomes.

Supplementary Table S4. Results of Radiological Outcomes.

bioengineering MDPI

Supplementary Table S1. Preferred Reporting Items for Systematic reviews and Meta-Analyses (PRISMA) checklist.

Section and Topic	Item #	Checklist item	Location where item is reported
**TITLE**			
Title	1	Identify the report as a systematic review.	Page 1
**ABSTRACT**			
Abstract	2	See the PRISMA 2020 for Abstracts checklist.	Page 1
**INTRODUCTION**			
Rationale	3	Describe the rationale for the review in the context of existing knowledge.	Page 2
Objectives	4	Provide an explicit statement of the objective(s) or question(s) the review addresses.	Page 2
**METHODS**			
Eligibility criteria	5	Specify the inclusion and exclusion criteria for the review and how studies were grouped for the syntheses.	Page 3
Information sources	6	Specify all databases, registers, websites, organisations, reference lists and other sources searched or consulted to identify studies. Specify the date when each source was last searched or consulted.	Page 3
Search strategy	7	Present the full search strategies for all databases, registers and websites, including any filters and limits used.	Page 3
Selection process	8	Specify the methods used to decide whether a study met the inclusion criteria of the review, including how many reviewers screened each record and each report retrieved, whether they worked independently, and if applicable, details of automation tools used in the process.	Page 3
Data collection process	9	Specify the methods used to collect data from reports, including how many reviewers collected data from each report, whether they worked independently, any processes for obtaining or confirming data from study investigators, and if applicable, details of automation tools used in the process.	Page 3-4
Data items	10a	List and define all outcomes for which data were sought. Specify whether all results that were compatible with each outcome domain in each study were sought (e.g. for all measures, time points, analyses), and if not, the methods used to decide which results to collect.	Page 3
	10b	List and define all other variables for which data were sought (e.g. participant and intervention characteristics, funding sources). Describe any assumptions made about any missing or unclear information.	Page 3-4
Study risk of bias assessment	11	Specify the methods used to assess risk of bias in the included studies, including details of the tool(s) used, how many reviewers assessed each study and whether they worked independently, and if applicable, details of automation tools used in the process.	Page 4
Effect	12	Specify for each outcome the effect measure(s) (e.g. risk ratio, mean difference) used in the synthesis or presentation of	Page 4

Section and Topic	Item #	Checklist item	Location where item is reported
measures		results.	
Synthesis methods	13a	Describe the processes used to decide which studies were eligible for each synthesis (e.g. tabulating the study intervention characteristics and comparing against the planned groups for each synthesis (item #5)).	Page 4
	13b	Describe any methods required to prepare the data for presentation or synthesis, such as handling of missing summary statistics, or data conversions.	Page 4
	13c	Describe any methods used to tabulate or visually display results of individual studies and syntheses.	Page 4
	13d	Describe any methods used to synthesize results and provide a rationale for the choice(s). If meta-analysis was performed, describe the model(s), method(s) to identify the presence and extent of statistical heterogeneity, and software package(s) used.	Page 4
	13e	Describe any methods used to explore possible causes of heterogeneity among study results (e.g. subgroup analysis, meta-regression).	Page 4
	13f	Describe any sensitivity analyses conducted to assess robustness of the synthesized results.	Not applicable
Reporting bias assessment	14	Describe any methods used to assess risk of bias due to missing results in a synthesis (arising from reporting biases).	Not applicable
Certainty assessment	15	Describe any methods used to assess certainty (or confidence) in the body of evidence for an outcome.	Not applicable
**RESULTS**			
Study selection	16a	Describe the results of the search and selection process, from the number of records identified in the search to the number of studies included in the review, ideally using a flow diagram.	Page 4<br>Figure 1
	16b	Cite studies that might appear to meet the inclusion criteria, but which were excluded, and explain why they were excluded.	Page 4
Study characteristics	17	Cite each included study and present its characteristics.	Page 5<br>Table 1-3
Risk of bias in studies	18	Present assessments of risk of bias for each included study.	Page 5<br>Table 4
Results of individual studies	19	For all outcomes, present, for each study: (a) summary statistics for each group (where appropriate) and (b) an effect estimate and its precision (e.g. confidence/credible interval), ideally using structured tables or plots.	Figure 2-6
Results of syntheses	20a	For each synthesis, briefly summarise the characteristics and risk of bias among contributing studies.	Page 5-7
	20b	Present results of all statistical syntheses conducted. If meta-analysis was done, present for each the summary estimate and its precision (e.g. confidence/credible interval) and measures of statistical heterogeneity. If comparing groups, describe the	Page 5-6<br>Figure 2-

Section and Topic	Item #	Checklist item	Location where item is reported
		direction of the effect.	6
	20c	Present results of all investigations of possible causes of heterogeneity among study results.	Page 5 Figure 3
	20d	Present results of all sensitivity analyses conducted to assess the robustness of the synthesized results.	Not applicable
Reporting biases	21	Present assessments of risk of bias due to missing results (arising from reporting biases) for each synthesis assessed.	Not applicable
Certainty of evidence	22	Present assessments of certainty (or confidence) in the body of evidence for each outcome assessed.	Not applicable
**DISCUSSION**			
Discussion	23a	Provide a general interpretation of the results in the context of other evidence.	Page 7
	23b	Discuss any limitations of the evidence included in the review.	Page 8-9
	23c	Discuss any limitations of the review processes used.	Page 8-9
	23d	Discuss implications of the results for practice, policy, and future research.	Page 9
**OTHER INFORMATION**			
Registration and protocol	24a	Provide registration information for the review, including register name and registration number, or state that the review was not registered.	Page 3
	24b	Indicate where the review protocol can be accessed, or state that a protocol was not prepared.	Page 3
	24c	Describe and explain any amendments to information provided at registration or in the protocol.	No
Support	25	Describe sources of financial or non-financial support for the review, and the role of the funders or sponsors in the review.	Page 10
Competing interests	26	Declare any competing interests of review authors.	Page 10
Availability of data, code and other materials	27	Report which of the following are publicly available and where they can be found: template data collection forms; data extracted from included studies; data used for all analyses; analytic code; any other materials used in the review.	Page 10

From: Page MJ, McKenzie JE, Bossuyt PM, Boutron I, Hoffmann TC, Mulrow CD, et al. The PRISMA 2020 statement: an updated guideline for reporting systematic reviews. BMJ 2021;372:n71. doi: 10.1136/bmj.n71

For more information, visit: http://www.prisma-statement.org/

Supplementary Table S2. Results of Anterior Knee Laxity.

year	author	Mean±SD of PRP	N of PRP	Mean±SD of Non-PRP	N of Non-PRP	Follow-up (months)	instruments	P value
2005	Ventura	0.8±1.4	10	1.2±0.9	10	6	KT-1000	NS
2009	Nin	0.5±1.375	50	0.5±1.25	50	24	KT-1000	NS
2010	Vogrin	4.9±1.8	22	6.1±2.1	23	3	KT-2000	**0.035**
2010	Vogrin	4.7±1.9	22	6.7±2.1	23	6	KT-2000	**0.003**
2013	Mirzatolooei	1.1±0.9	23	2.2±1.1	23	3	KT-1000	**< 0.001**
2013	Vadála	2.9±1.2	20	2.8±3.1	20	12	KT-1000	NS
2014	Starantzis	3.72±0.54	25	3.69±0.74	26	12	Rolimeter	0.879
2022	Gong	0.85±0.99	27	1.39±1.98	26	12	KT-2000	0.62
2024	Lin	1.3±1.1	8	1±0.5	10	3	KT-1000	NS
2024	Lin	1±1.2	8	1.4±0.6	10	6	KT-1000	NS
2024	Lin	1±0.9	8	1.2±0.5	10	12	KT-1000	NS

SD, Standard Deviation; N, number; PRP, Platelet-Rich Plasma; NS, Not Significant

Supplementary Table S3. Results of Other Comparable Continuous Outcomes.

Author-Year	Outcomes	PRP Group			Non-PRP Group			P-value	Follow-up
		Mean	SD	N	Mean	SD	N		
Ventura-2005	KOOS	83	20	10	84	9	10	NS	6 months
Ye-2024	$KOOS_4$	78.3	12	57	76.8	11.9	57	0.36	12 months
	KOOS-pain	77.4	14.1	59	78.7	16.1	58	0.85	3 months
		86.3	10.6	59	83.6	10.5	57	0.07	6 months
		88.9	9	57	87.6	9.1	57	0.26	12 months
	KOOS-symptoms	60.6	17.2	59	60.8	19	58	0.83	3 months
		72.6	17.9	59	68.4	18.8	57	0.13	6 months
		76.5	13.5	57	77.4	14.8	57	0.86	12 months
	KOOS-ADL	87.6	11.7	59	87.7	11.8	58	0.94	3 months
		94.8	6.6	59	92.3	9	57	0.10	6 months
		96.7	4.7	57	94.9	7	57	0.15	12 months
	KOOS-SR	48.3	26.1	59	49.1	26	58	0.94	3 months
		72.9	18.9	59	66.9	19.1	57	**0.046**	6 months
		81.1	16.1	57	79.8	15.4	57	0.58	12 months
	KOOS-QoL	41.4	20.3	59	44.4	22	58	0.47	3 months
		56	20.5	59	50.1	21.1	57	0.10	6 months
		66.8	19.9	57	62.4	19.1	57	0.19	12 months
Cervellin-2012	VISA	97.8	2.5	20	84.5	11.8	20	**< 0.05**	12 months
de Almeida-2012	Kujala	86.25	8.56	12	84.75	6.61	15	0.33	6 months
	IS	30.6	21.9	12	30.9	10.8	15	0.57	6 months
Gong-2022	Proprioception	2.24	0.83	30	2.26	0.81	29	0.92	3 months
		2.67	1.23	29	2.45	0.93	26	0.65	6 months
		2.8	0.7	27	2.69	0.86	26	0.39	12 months

SD, Standard Deviation; N, number; KOOS, Knee Injury and Osteoarthritis Outcome Score; $KOOS_4$, 4 subscales of the Knee Injury and Osteoarthritis Outcome Score; ADL, Activity of Daily Living; SR, Sport and Recreation; QoL, Quality of Life; VISA, Victorian Institute Sport Assessment Scale; IS, Isokinetic Strength; NS, Not Significant.

Supplementary Table S4. Results of Radiological Outcomes.

Outcome	Author	Instrument	Definition of the radiological outcomes	Findings	Follow-up (months)	PRP group	Non-PRP group	P-value
graft maturation and integration	Ventura	CT	ACL density expressed as Huasfield scale	ACL density of PRP group was significant lower than non-PRP group and densities of the ACL and PCL were similar in PRP group.	6	82.72±9.57	95.11±7.24	**p < 0.01**
	Orrego	MRI	graft signal intensity at the femoral tunnel: low-intensity signal equivalent to the PCL vs high-intensity signal similar to the synovial fluid	The graft signal intensity of PRP group was no different from non-PRP group at 3 month but significantly lower than non-PRP group at 6 month.	3	46% vs 54%	44% vs 56%	p = 0.14
					6	100% vs 0%	78% vs 22%	**p = 0.04**
			absence of osteoligamentous interface between the graft and bone at the femoral tunnel	No statistical differences were observed regarding the osteoligamentous interface between the groups both at 3- and 6-month.	3	42% vs 58%	44% vs 56%	p = 0.59
					6	88% vs 12%	67% vs 33%	p = 0.11
	Nin	MRI	intensity measured with an ROI at the center of the graft	The results did not show any statistically significant differences between the groups for intensity measured with an ROI	6	proton density-weighted images: 230 T2-weighted images: 75	proton density-weighted images: 190 T2-weighted images: 61	proton density-weighted images: p = 0.45 T2-weighted images: p = 0.10
	Silva	MRI	signal intensity of fibrous interzone in the posterolateral femoral tunnels on PDW-FatSat: grade 1 similar to skeletal muscle vs grade 2 greater than muscle but less than fluid	There were not any differences between the groups when comparing the signal intensity in the fibrous interzone of the femoral tunnels on MRI.	3	67% vs 33%	90% vs 10%	
			signal intensity of fibrous interzone in the anteromedial femoral tunnels on PDW-FatSat: grade 1 similar to skeletal muscle vs grade 2 greater than muscle but less than fluid		3	67% vs 33%	80% vs 20%	
			signal intensity of fibrous interzone in the posterolateral femoral tunnels on		3	17% vs 80% vs 3%	20% vs 80%	

		T1W-FatSat-Gad: grade 1 similar to skeletal muscle vs grade 2 greater than muscle but less than synovial membrane vs grade 3 similar to synovial membrane					
		signal intensity of fibrous interzone in the anteromedial femoral tunnels on T1W-FatSat-Gad: grade 1 similar to skeletal muscle vs grade 2 greater than muscle but less than fluid		3	27% vs 70% vs 3%	10% vs 90%	
Vogrin	MRI	vascularization rate defined as the difference in the SNQ after and before the application of the contrast medium for each ROI in the interface zone between the graft and tibial tunnel	At 4-6 weeks, vascularization levels were significantly higher in the PRP group than non-PRP group in the interface zone between the graft and tibial tunnel, but this significant difference disappeared at 10 to 12 weeks.	1 to 1.5	0.33±0.09	0.16±0.09	**p < 0.001**
				2.5 to 3	0.20±0.13	0.17±0.10	p = 0.40
		vascularization rate defined as the difference in the SNQ after and before the application of the contrast medium for each ROI in the intra-articular part of the graft	No statistical differences were observed regarding the vascularization rate in the intra-articular part of the graft between the groups at both times.	1 to 1.5	–0.01±0.08	0.01±0.02	p = 0.26
				2.5 to 3	0.01±0.06	0.02±0.04	p = 0.40
Seijas	MRI	remodelling stages of the grafts: ashypointense (same intensity as PCL) vs mildly hyperintense (<1/3 hyperintense zones) vs moderately hyperintense (<2/3 hyperintense zones)vs severely hyperintense (>2/3 hyperintense zones) vs diffusely hyperintense (same intensity as PCL)	More patients in the PRP group achieved higher stages of remodeling than non-PRP group, with a statistically significant difference in month 4 and 6, but no statistically significant difference in month 12.	4	8% vs 12% vs 41% vs 25% vs 14%	14% vs 39% vs 25% vs 18% vs 4%	**p = 0.003**
				6	2% vs 4% vs 18% vs 21% vs 45%	12% vs 22% vs 21% vs 29% vs 6%	**p = 0.0001**
				12	0% vs 2% vs 2% vs 27% vs 69%	0% vs 4% vs 4% vs 31% vs 61%	p = 0.35
Rupreht	MRI	cellularity of the graft: ADC maps were calculated from the two DWI image sets of different b values (0 s/mm2 and 400s/mm2) using the MRI Analysis Calculator plugin of the ImageJ, higher ADC values indicate lower cellularity.	At 1 month, the average ADC value in the PRP group was significantly lower than in the non-PRP group, demonstrating a reduced random motion of water in the ROI in the PRP group. At the 2.5- and 6-month, ADC values were non significantly lower in the PRP group.	1	1.41	1.5	**p = 0.03**
				2.5	1.32	1.37	p = 0.42
				6	1.21	1.29	p = 0.11

		vascularization in the interface zone and the graft: Genh = 100((SImax-SI0)/(SI0τ)), indicates the signal increase of contrast medium per unit time, higher values indicate better vascularization.	At 1 and 2.5 months average Genh value was significantly higher in the PRP group than non-PRP group. At 6 months, there was no significant difference in average Genh between the groups.	1	2.07	1.41	**p = 0.02**
				2.5	1.64	1.15	**p < 0.01**
				6	0.93	0.84	p = 0.53
		vascularization in the interface zone and the graft:Fenh = (SImax-SI0)/SI0, indicates the amount of contrast accumulated in ROI compared with the baseline,higher values indicate better vascularization.	At 1, 2.5, and 6 months, average Fenh was higher in the PRP group than in the non-PRP group, although not statistically significantly.	1	1.36	1.16	p = 0.10
				2.5	1.02	0.88	p = 0.07
				6	0.66	0.62	p = 0.42
Rupreht	MRI	Tunnel wall cortical bone was defined as a clearly hypointense rim of the tibial tunnel wall that was at least 1 mm thick. The portion of the tunnel wall circumference, consisting of TCB, was rounded off to ten percent.	At 1 month we found nonsignificant difference between the groups. At 2.5 and 6 months, the percentage of TCB was significantly higher in the PRP group than in the non-PRP group.	1	6.7 (1.8-11.5)	5.0 (1.8-8.2)	p = 0.93
				2.5	36.2 (28.7-43.7)	22.5 (17.3-27.7)	**p = 0.0004**
				6	67.1 (61.0-73.3)	53.5 (47.0-60.0)	**p = 0.0003**
Mahdi	MRI	signal intensity of fibrous interzone of femoral tunnel on MRI signal grade PDW-Fat Sat: grade 0 palelar tendon vs grade 1 skeletal fousele	PRP group achieved better graft maturity than non0PRP group, although the difference was not statistically significant.	3+	29% vs 71%	0% vs 100%	p = 0.057
		signal intensity of fibrous interzone of femoral tunnel on MRI signal grade TIW-Fat Sat-Gad: grade1 palelar tendon vs grade 2 skeletal fousele vs grade 3 >Skeletal Muscle < synovial membrane		3+	14% vs 86%	0% vs 85% vs 15%	p = 0.067
Gong	MRI	SNQ of ROI intra-articular graft to evaluate graft maturation	At 12-month, SNQs for the FT (tendon–bone healing) and IAG (graft maturation) in the PRP group were lower than those of the non-PRP group, but the diferences were not signifcant.	12	1.38±0.70	2.01±0.62	p = 0.06
		SNQ of ROI femoral tunnel graft to evaluate tendon–bone healing		12	2.39±1.22	2.46±0.83	p = 0.89
Munde	MRI	synovial fluid at graft tunnel interface: absent vs present	PRP group had significantly more patients without synovial fluid at the graft tunnel interface than non-PRP group.	6	58% vs 42%	35% vs 65%	**p = 0.04**

		graft intensity compared with semimembranosus muscle: hyperintense vs isointense vs hypointense	The values were not significantly different between the two groups.	6	40% vs 18% vs 42%	53% vs 5% vs 42%	p = 0.17
		Figueroa's scores of > 3 were considered indicative of adequate graft maturation: > 3 vs ≤ 2	Significantly more patients in PRP group achieved graft maturity than in non-PRP group.	6	80% vs 20%	65% vs 35%	**p = 0.019**
Lin	MRI	The graft maturation was recorded as graft signal intensity. The whole graft signal (proximal, middle, and distal portions of the ACL graft) was averaged and normalised by individual PCL signal (A/P ratio).	There were no signifcant diferences between the PRP and non-PRP groups at the four time points.	1.5	3.57±0.47	4.24±0.51	p > 0.05
				3	4.39±0.40	5.51±0.51	p > 0.05
				6	4.58±0.37	4.54±0.27	p > 0.05
				12	5.13±0.85	4.35±0.51	p > 0.05
		change of peri-femoral tunnel edema by dividing the measured peri-tunnel signal with the signal of bone marrow in the middle third of the femoral shaft.		1.5	2.75±0.31	2.67±0.30	p > 0.05
				3	1.81±0.15	2.00±0.16	p > 0.05
				6	1.50±0.19	1.43±0.07	p > 0.05
				12	1.44±0.15	1.22±0.08	p > 0.05
		change of peri-tibial tunnel edema by dividing the measured peri-tunnel signal with the signal of bone marrow in the middle third of the femoral shaft.		1.5	3.49±0.47	3.54±0.24	p > 0.05
				3	2.71±0.51	2.47±0.29	p > 0.05
				6	1.98±0.15	2.06±0.22	p > 0.05
				12	1.96±0.22	1.84±0.20	p > 0.05
Ye	MRI	femoral intratunnel segment of anteromedial bundle: SNQ(signal intensity of ACL graft-signal intensity of quadriceps tendon)/signal intensity of background	The PRP group demonstrated improved graft maturity at 6 months in the intra-articular and femoral intratunnel segments of both the anteromedial bundle and the posterolateral bundle compared with the non-PRP group. No statistically significant between-group differences were found for graft maturity on the 3- and 12-month follow-up.	3	9.2 (5.7-15.6)	11.5 (4.2-16.6)	p = 0.95
				6	13.4 (10.2-17.7)	17.4 (12.8-21.5)	**p = 0.003**
				12	9.8 (6.1-17.4)	12.3 (8.1-17.7)	p = 0.12
		intra-articular segment of anteromedial bundle: SNQ(signal intensity of ACL graft-signal intensity of quadriceps tendon)/signal intensity of background		3	6.6 (4.2-11.0)	6.2 (3.4-10.2)	p = 0.54
				6	10.3 (5.9-13.8)	13.6 (8.7-17.6)	**p = 0.03**
				12	8.1 (5.6-11.4)	9.5 (5.1-13.6)	p = 0.38
		tibial intratunnel segment of anteromedial bundle: SNQ(signal intensity of ACL graft-signal intensity of quadriceps tendon)/signal intensity of background		3	6.0 (2.3-9.7)	4.4 (2.1-8.0)	p = 0.26
				6	10.2 (6.4-13.6)	9.5 (5.7-14.7)	p = 0.90
				12	8.7 (5.9-11.4)	8.1 (5.0-11.7)	p = 0.63
		femoral intratunnel segment of posterolateral bundle: SNQ(signal intensity of ACL graft-signal intensity of quadriceps tendon)/signal intensity of background		3	9.3 (4.4-14.5)	11.3 (5.2-17.3)	p = 0.40
				6	15.4 (10.9-21.0)	19.6 (14.2-24.1)	**p = 0.02**
				12	10.4 (6.2-19.9)	13.8 (8.8-20.3)	p = 0.19

			intra-articular segment of posterolateral bundle: SNQ(signal intensity of ACL graft-signal intensity of quadriceps tendon)/signal intensity of background		3	8.4 (4.8-12.9)	7.5 (3.8-12.6)	p = 0.48
					6	11.4 (7.1-17.4)	15.1 (11.2-19.5)	**p = 0.007**
					12	7.9 (5.5-11.8)	10.2 (5.8-14.6)	p = 0.16
			tibial intratunnel segment of posterolateral bundle: SNQ(signal intensity of ACL graft-signal intensity of quadriceps tendon)/signal intensity of background		3	6.0 (3.4-9.8)	5.1 (2.7-8.3)	p = 0.41
					6	11.1 (6.1-16.6)	10.1 (5.9-16.5)	p = 0.81
					12	7.6 (5.3-10.9)	7.2 (4.0-11.4)	p = 0.94
tunnel widening	Orrego	MRI	absence of femoral tunnel widening: a difference greater than 2 mm between the 3 and 6 month measurements was considered to be a widened tunnel.	No significant difference in tunnel widening was observed between the two groups 3 to 6 months after surgery	3 to 6	69% vs 31%	59% vs 41%	p = 0.45
	Mirzatolooei	CT	widening of the femoral tunnel opening between the day after operation and three months later	Despite slightly less tunnel widening in the PRP group, there were no significant differences at any of the sites of measurement between immediately after surgery and three months post-operatively.	0 to 3	2.16±1.37	2.50±1.22	p = 0.44
			widening of the mid-femoral tunnel between the day after operation and three months later		0 to 3	2.69±2.06	3.21±1.72	p = 0.42
			widening of the tibial tunnel opening between the day after operation and three months later		0 to 3	1.65±0.98	1.99±1.31	p = 0.23
			widening of the mid-tibial tunnel between the day after operation and three months later		0 to 3	2.09±1.52	2.70±1.48	p = 0.21
	Vadala	CT	femoral tunnel diameters (mm) measured at four different levels for tibial and femoral tunnel	Even though a statistical difference was always found comparing pre- and post-operative bone tunnel diameters for femoral and tibial tunnels in all patients of both groups, comparison of final values between the two groups never showed statistically significant differences.	0	9.0±0.1	9.1±0.1	p > 0.05
			femoral tunnel diameters (mm) measured at four different levels for tibial and femoral tunnel		14.7(10-16)	9.8±0.3	9.4±0.5	p > 0.05
			tibial tunnel diameters (mm) measured at four different levels for tibial and femoral tunnel		0	9.0±0.2	9.1 ± 0.1	p > 0.05
			tibial tunnel diameters (mm) measured at four different levels for tibial and femoral tunnel		14.7(10-16)	10.9±0.2	10.1±0.4	p > 0.05
	Starantzis	MRI	diameters (mm) measured at the proximal of the femoral tunnel		0	82.40±4.59	80.19±5.91	p = 0.14
					1	83.64±5.11	81.19±6.43	p = 0.14

					12	87.52±8.26	88.35±11.43	p = 0.78
			diameters (mm) measured at the mid-distance of the femoral tunnel	The tunnel diameter between the 2 groups during the observation period was not significantly different.	0	82.40±4.59	80.19±5.91	p = 0.14
					1	83.32±4.63	80.81±6.41	p = 0.12
					12	86.32±7.75	87.50±9.63	p = 0.63
			diameters (mm) measured at the distal of the femoral tunnel		0	82.40±4.59	80.19±5.91	p = 0.14
					1	83.04±4.67	80.65±6.39	p = 0.14
					12	83.52±6.46	83.65±8.00	p = 0.95
	Gong	CT	femoral tunnel diameters (mm)	There were no signifcant diferences between the study and control groups at the two time points.	0	6.91±0.74	7.30±1.17	p > 0.05
			femoral tunnel diameters (mm)		12	8.88±1.46	8.42±2.75	p > 0.05
			tibial tunnel diameters (mm)		0	9.31±0.83	9.36±0.88	p > 0.05
			tibial tunnel diameters (mm)		12	9.50±1.07	9.99±1.91	p > 0.05
	Kumar	X-ray	tibial tunnel width (mm)	There were no signifcant diferences between the study and control groups at the three time points.	0	8.54±0.61	8.51±0.51	p > 0.05
					1.5	8.83±0.62	8.79±0.50	p > 0.05
					3	9.12±0.60	9.05±0.52	p > 0.05
	Lin	MRI	change of femoral bone tunnel diameter by dividing the measured bone tunnel diameter with the original bone tunnel diameter	There were no signifcant diferences between the PRP and non-PRP groups at the four time points.	1.5	1.43±0.04	1.36±0.04	p > 0.05
					3	1.49±0.06	1.47±0.04	p > 0.05
					6	1.48±0.04	1.51±0.04	p > 0.05
					12	1.50±0.07	1.48±0.06	p > 0.05
			change of tibial bone tunnel diameter by dividing the measured bone tunnel diameter with the original bone tunnel diameter		1.5	1.43±0.05	1.38±0.05	p > 0.05
					3	1.44±0.05	1.45±0.06	p > 0.05
					6	1.51±0.05	1.47±0.03	p > 0.05
					12	1.49±0.05	1.51±0.03	p > 0.05
harvest site healing	Cervellin	MRI	satisfactory bone filling: a filling of 70% of the bone gap at the harvesting site	Tibial and patellar defects were satisfactorily filled with new bone tissue (covered area >70%) in 80% of patients in the PRP group, compared with 60% in the control group.	12	80% vs 20%	60% vs 40%	p > 0.05
	de Almeida	MRI	gap area of the patellar tendon harvest site (mm2): Measurement was performed at the midpoint through the length of the tendon from the apex of the patella to the insertion at the tibial tubercle.	Patellar tendon harvest site nonregenerated area (gap area) of PRP group was significantly lower than the control group.	6	4.9±5.4	9.4± 4.4	**p = 0.046**
	Seijas	US	repair process at the donor site: A represented the absence of repair vs B a start in the repair process with less than 50 % scar tissue vs C advanced repair process with more than 50 % repaired tissue vs D complete repair	Individuals with PRP were more associated with higher scores of maturity than individuals without PRP at 4 month. No differences at the other follow-up times were found.	1	70% vs 30% vs 0% vs 0%	84.2 15.8 0.0 0.0	p > 0.05
					2	39% vs 26% vs 35% vs 0%	63.2 10.5 26.3 0.0	p > 0.05
					4	4% vs 26% vs 18% vs 52%	10.5 68.4 10.5 10.5	**p = 0.0037**

				6	0% vs 9% vs 13% vs 78%	0.0 10.5 26.3 63.2	p > 0.05
				9	0.0 0.0 4.4 95.7	0.0 5.3 5.3 89.5	p > 0.05
				12	0.0 0.0 0.0 100	0.0 0.0 0.0 100	p > 0.05
Walters	MRI	the largest transverse signal defect (mm) in normal bone marrow of the patella at the graft site	The transverse width of the osseous defect at the largest patellar graft site was not different between groups.	6 to 9	11.6±2.4	12.0±3.1	p = 0.53

Disclaimer/Publisher's Note: The statements, opinions and data contained in all publications are solely those of the individual author(s) and contributor(s) and not of MDPI and/or the editor(s). MDPI and/or the editor(s) disclaim responsibility for any injury to people or property resulting from any ideas, methods, instructions or products referred to in the content.
